# Hip arthroscopy with initial access to the peripheral compartment for femoroacetabular impingement: midterm results from a large-scale patient cohort

**DOI:** 10.1186/s10195-024-00770-6

**Published:** 2024-05-24

**Authors:** Moritz Wagner, Richard A. Lindtner, Luca Schaller, Florian Schmaranzer, Ehrenfried Schmaranzer, Peter Vavron, Franz Endstrasser, Alexander Brunner

**Affiliations:** 1 Department of Orthopaedics and Traumatology, District Hospital St. Johann in Tirol, Bahnhofstrasse 14, 6380 St. Johann in Tirol, Austria; 2 Department of Orthopaedics and Traumatology, Paracelsus Medical University, Salzburg, Austria; 3 Medical University of Innsbruck, Anichstrasse 35, 6020 Innsbruck, Tyrol Austria; 4 Department of Orthopaedics and Traumatology, Medical University of Innsbruck, Anichstrasse 35, 6020 Innsbruck, Tyrol Austria; 5 Department of Radiology, Inselspital, Universitätsspital Bern, Bern, Switzerland; 6 Department of Radiology, District Hospital St. Johann in Tirol, Bahnhofstrasse 14, 6380 St. Johann in Tirol, Austria

**Keywords:** Hip joint, Arthroscopy, Surgical technique, Peripheral compartment, Femoroacetabular impingement, Outcome

## Abstract

**Background:**

Hip arthroscopy with initial access to the peripheral compartment could reduce the risk of iatrogenic injury to the labrum and cartilage; furthermore, it avoids the need for large capsulotomies with separate portals for peripheral and central (intra-articular) arthroscopy. Clinical results of the peripheral-compartment-first technique remain sparse, in contrast to those of conventional hip arthroscopy starting in the intra-articular central compartment. The purpose of this study was to assess outcome of hip arthroscopy with the peripheral-compartment-first technique, including complication rates, revision rates and patient-reported outcome scores.

**Materials and methods:**

This outcome study included 704 hips with femoroacetabular impingement. All arthroscopies were performed using the peripheral-compartment-first technique. A joint replacement registry and the institutional database were used to assess the revision and complication rates, while patient-reported outcome measures were used to assess functional outcomes and patient satisfaction.

**Results:**

In total, 704 hips (615 patients) were followed up for a mean of 6.2 years (range 1 to 9 years). The mean age of the patients was 32.1 ± 9.2 years. During the follow-up period, 26 of 704 (3.7%) hips underwent total hip arthroplasty (THA) after a mean of 1.8 ± 1.2 years, and 18 of the 704 (2.6%) hips required revision hip arthroscopy after a mean of 1.2 ± 2.1 years. 9.8% of the hips had an unsatisfactory patient-reported outcome at final follow-up.

**Conclusions:**

The results for the peripheral-compartment-first technique were promising. We recommend a well-conducted randomized controlled clinical trial to guide future therapeutic recommendations regarding the most favorable hip arthroscopy technique.

*Level of evidence:* Level IV, therapeutic study.

*Trial registration:* This study was registered at ClinicalTrials.gov (U.S. National Library of Medicine; ID: NCT05310240).

## Introduction

Hip arthroscopy is a well-established surgical procedure for the treatment of femoroacetabular impingement (FAI) with reliable symptom relief, which is evident from the significant improvement in patient-reported outcome scores [1]. In addition, it is associated with a low complication rate (1.7%) [2] and a moderate conversion rate to total hip arthroplasty (3.0 to 17.9%), as shown by a recent systematic review [3]. Controversy exists regarding the most favorable surgical technique [4]. The most frequently used surgical technique is to place portals under traction, starting with the central (intra-articular) compartment. Fluoroscopy and the surgeon’s tactile feeling are used to guide intra-articular portal placement and limit damage to the cartilage and labrum; however, iatrogenic cartilage damage is still one of the most frequently reported complications after hip arthroscopy [5]. Hip arthroscopy with initial access to the central compartment usually involves a capsulotomy between the peripheral and central compartments to enable direct access to both compartments [6]. The majority of the literature on outcomes after hip arthroscopy report on surgeries that use this ‘traditional’ technique [2].

Hip arthroscopy with initial access to the peripheral compartment could reduce the risk of iatrogenic injury to the labrum and the cartilage, avoiding the need for large capsulotomies with separate portals for peripheral and central (intra-articular) arthroscopy. To avoid damage to the intra-articular chondral surface, the arthroscope may be first inserted into the peripheral compartment and onto the femoral neck [7]. The peripheral compartment is approached without traction, with the hip slightly flexed and internally rotated to protect the femoral head. The second step involves access to the central portal, with direct visualization. The same skin incisions may be used for different portals; the joint capsule between portals does not have to be incised—separate stabbing incisions are used. To precisely guide secondary intra-articular portal placement, a needle is advanced under direct arthroscopic visualization from the peripheral compartment. This portal placement technique allows capsule-preserving stab incisions and avoids the need for a capsulotomy. Portals may be enlarged, and the joint capsule may be mobilized from the bone, but no peri-portal capsulotomy is performed. Minimal capsulotomies allow for better intra-capsular fluid retention, creating a ballooning effect. The visual guidance during intra-articular portal placement is, furthermore, intended to reduce the risk of iatrogenic injury to the labrum and the cartilage and the risk for traction-related morbidity, owing to the reduced traction time. This technique has been described in more detail, with informative illustrations, in a publication from Tang, Brockwell and Dienst [6]. Until now, only a few studies have reported clinical outcomes for the peripheral-compartment-first technique with a short-term follow-up period [8–11].

Clinical results of the peripheral-compartment-first technique remain sparse, in contrast to those of conventional hip arthroscopy starting in the intra-articular compartment. The purpose of this study was (1) to evaluate revision and complication rates as well as patient-reported outcome measures of hip arthroscopy starting in the peripheral compartment and (2) to compare these results with those previously reported for hip arthroscopy starting in the central compartment.

## Materials and methods

**Fig. 1:**
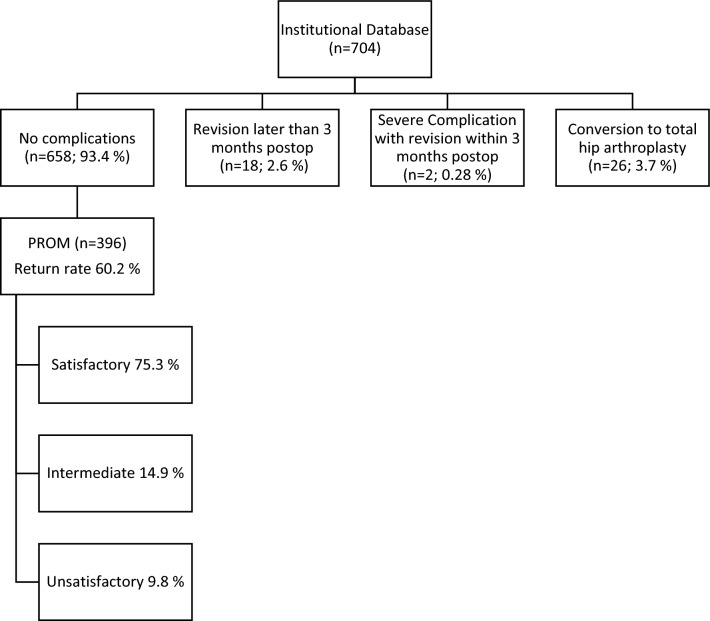
Flowchart of patient recruitment according to the STROBE recommendations

This registered cohort study (ClinicalTrials.gov ID: NCT05310240) was conducted at a single center (Department of Orthopaedics and Traumatology, District Hospital St. Johann in Tirol, St. Johann in Tirol, Austria) and included the data on patients who presented to the institution from January 2013 to April 2021. The study was approved by the ethics committee of the Medical University of Innsbruck, Austria (approval no. 1199/2021). All patients who underwent hip arthroscopy during this period were included. A total of 810 hip arthroscopies were identified from the electronic institutional database. Patients receiving hip arthroscopy for any other condition than FAI were excluded, resulting in 704 hips (in 615 patients) (Fig. 1).

**Fig. 2:**
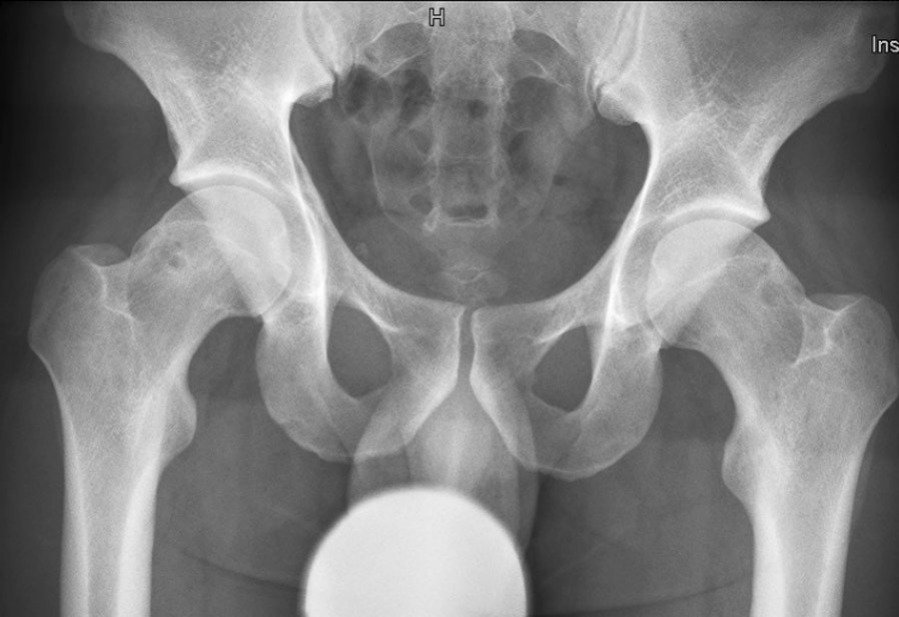
Routine preoperative radiograph showing a bilateral CAM deformity

**Fig. 3:**
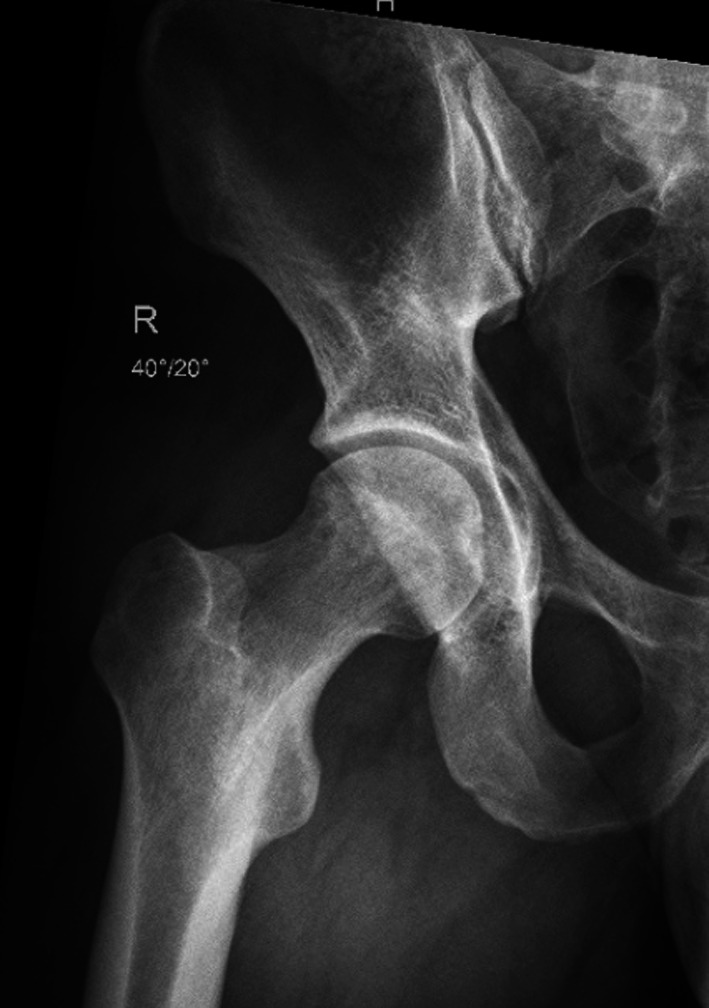
Routine postoperative radiograph with a modified Dunn view following right-sided femoral osteoplasty for the same patient

All patients underwent thorough imaging, including standard conventional radiography with anterior–posterior pelvis (Fig. 2), modified Dunn and false-profile views as well as magnetic resonance arthrography with traction followed by evaluation by a specialist musculoskeletal radiologist [12, 13]. After surgery, the modified Dunn radiograph was repeated following femoral osteoplasty (Fig. 3). In cases with residual pain and a limited range of motion after the procedure, repeated magnetic resonance imaging (MRI) was performed to assess the remaining pathologies and to plan revision hip arthroscopy if necessary. All arthroscopies were carried out by two experienced surgeons, starting in the peripheral compartment [7]. Patients underwent a variety of procedures, including bony resection of the head–neck junction in patients with a CAM-type deformity, trimming of the acetabular rim together with labral repairs in patients with a pincer-type deformity, and isolated labral repair with only minimal trimming of the acetabular rim to promote healing from cancellous bone.

During the procedure, patients were placed in a supine position using a well-padded perineal post and a standard manual traction table. The first arthroscopic portal for the peripheral compartment was placed under fluoroscopic control, with slight internal rotation of the leg to protect the femoral cartilage and acetabular labrum [7]. The results of the femoral osteoplasty for CAM deformity were routinely assessed with intraoperative fluoroscopy [14, 15]. Labral repairs were performed in a standardized fashion. Labral damage was assessed with a small arthroscopic hook. In the case of labral instability, the labrum was partially detached from the acetabular rim. In the case of over-coverage of the acetabulum, the acetabular rim was trimmed down with an oscillating burr. The labral repair was then performed with trans-labral sutures using 2.9-mm and/or 3.5-mm intra-osseous push-lock anchors (PushLock® Anchor; Arthrex, Naples, FL, USA) with a toggle stitch configuration. Labral stability was then assessed intraoperatively. The number of suture anchors used varied depending on the extent of labral damage and the requirements for stability.

The postoperative rehabilitation protocol consisted of 4 weeks of partial weight-bearing for all patients after femoral osteoplasty and 6 weeks of limited hip flexion to 80° for all patients after labral repair. Deep venous thrombosis prophylaxis was performed with low-molecular-weight heparin for 6 weeks in all cases.

The clinical information on all patients was assessed, including diagnosis, details of the surgical intervention, duration of the procedure, duration of intraoperative radiation exposure, dosage of opioid analgesics during in-hospital stay, length of in-hospital stay, and complications leading to revision or re-hospitalization within 3 months. The joint replacement surgeries were identified with the help of an arthroplasty registry [16] with state-wide coverage and a capture rate of 97.9% for primary total hip arthroplasty (THA). The revision arthroscopies were identified using the institutional database.

The patient-reported outcome was assessed with a single questionnaire at final follow-up at a single time point. The modified Harris Hip Score (mHHS) [17], International Hip Outcome Tool (iHOT-12) [18] and the visual analog scale for pain (VAS) were assessed. A question regarding revision hip arthroscopy or joint replacement surgery at other institutions was included to compensate for missing institutional or registry data. An anchor question regarding patient satisfaction was included.

### Statistical analysis

Numerical parameters were checked for a normal distribution. The means of continuous variables were compared between sub-groups using one-way analysis of variance with Tukey’s post-hoc testing. Categorical data were compared using the chi-square test. Statistical significance was set at *p* ≤ 0.05 for all tests.

## Results

In total, 704 hips (615 patients) were followed up for a minimum of 1 to a maximum of 9 years (mean 6.2 ± 2.1 years). The mean age of the patients was 32.1 ± 9.2 years, and the majority of patients were male (428 of 704 hips, 60.9%). More arthroscopies (362 of 704 hips, 51.5%) were performed on the right side, whereas 184 of 704 (26.1%) arthroscopies (92 patients) were bilateral.

During the follow-up period, 26 of 704 (3.7%) hips underwent THA after a mean of 1.8 ± 1.2 years, and 18 of 704 (2.6%) hips required revision hip arthroscopy after a mean of 1.2 ± 2.1 years. A complication requiring revision surgery within 3 months occurred in two patients (2 of 704), resulting in a complication rate of 0.28%. Both patients suffered from stress edema around the femoral neck after femoral osteoplasty, which was evident on magnetic resonance imaging, mandating percutaneous screw fixation of the femoral neck.

The mean length of in-hospital stay was 2.4 ± 0.7 days. The mean set-up time in the operating room from the arrival of the patient and initiation of anaesthesia to patient preparation on the traction table with a trial traction was 38 ± 19 min. The mean duration of surgery from skin incision to complete wound closure was 108 ± 37 min. The mean time from complete wound closure to the patient leaving the operating room was 34 ± 24 min. The duration of intraoperative radiation exposure was 29 ± 24 s, resulting in a radiation dosage of 3.4 ± 2.6 mGy.

The questionnaire was completed for 396 of 658 (60.2%) hips. Of those, 75.3% reported satisfactory outcomes and would have undergone the surgery again retrospectively, 14.9% were unsure whether they would have undergone the surgery again, and 9.8% would not have undergone the surgery again (Fig. 1). This anchor question regarding patient satisfaction correlated strongly with a satisfactory patient-reported outcome for pain (*p* < 0.001), iHOT-12 (*p* < 0.001) and mHHS (*p* < 0.001). The mean pain score before surgery (recall bias) was 6.6 ± 2.5, which decreased to 2.8 ± 2.3 after surgery. The mean mHHS at follow-up was 86.2 ± 13.1. The mean iHOT-12 score at follow-up was 78.7 ± 21.8. Diagnoses were obtained from surgical reports and are listed in Table 1.

**Table 1:** Comparison of patient age, length of hospital stay (LOS) and patient-reported outcome between diagnoses

Diagnosis	Age (years)	LOS (days)	mHHS (100 is best, 0 is worst)	iHOT-12 (100 is best, 0 is worst)	VAS (0 is best, 100 is worst)
CAM FAI (*n* = 384)	35.5 ± (SD 10.4)	2.9 (SD 0.7)	87.0 (SD 12.5)	79.1 (SD 21.9)	26.1 (SD 22.3)
Mixed FAI (*n* = 182)	32.2 (SD 10.2)	3 (SD 0.7)	85.3 (SD 14.2)	77.3 (SD 22.8)	31.6 (SD 24.6)
Pincer FAI (*n* = 138)	32.1 (SD 10.7)	3.1 (SD 0.9)	85.2 (SD 13.6)	79.6 (SD 20.8)	30.2 (SD 24.7)
Total (*n* = 704)	32.1 (SD 9.2)	2.4 (SD 0.7)	86.2 (SD 13.1)	78.7 (SD 21.8)	28.3 (SD 23.4)

The data are given as the mean and standard deviation (SD)

*mHHS* modified Harris Hip Score, *iHOT-12* International Hip Outcome Tool with 12 questions, *VAS* visual analog scale for pain, *FAI* femoroacetabular impingement

A total of 329 (46.7%) hips underwent combined femoral osteoplasty and labral repair, 338 (43.0%) hips underwent isolated femoral osteoplasty, and 37 (5.2%) hips underwent isolated labral repair. In most cases, the labral repairs were performed with three suture anchors (mean 2.7 ± 0.8 anchors). Isolated femoral osteoplasty required a total of 1 h and 39 min. Isolated labral repair took only a few minutes longer, with an average of 1 h and 52 min. A combination of both procedures required 2 h and 14 min (*p* < 0.001). Patients who underwent isolated femoral osteoplasty were exposed to radiation for the longest duration (38 s), and those who underwent isolated labral repair were exposed for the shortest duration (9 s, *p* < 0.001). 

## Discussion

The overall results, measuring the complication rate, revision rate and patient-reported outcome, were in favor of the peripheral-compartment-first technique. Those results are in accordance with previously published studies [9, 10, 19]. In our patient cohort with a mean follow-up period of 6.2 ± 2.1 years, about 3.7% of patients converted to THA, only 2.6% of patients underwent revision hip arthroscopy, and as little as 0.28% of patients experienced a serious complication requiring fixation of the femoral neck. As little as 9.8% of the patients would not have this procedure performed again retrospectively.

In comparison with the results from a recent meta-analysis of 31 clinical studies, including a total of 1981 hips, the complication rate (0.28% vs. 1.7% [2]) was lower in this study. Moreover, conversion to total hip arthroplasty was less common in our series [26/704 hips (3.7%) vs. 128/1981 hips (6.5%)], although the mean follow-up period was substantially longer (6.2 ± 2.1 years vs. 29.5 ± 13.9 months). An even more recent systematic review reported an average conversion to THA rate of 11.1% (range 3.0 to 17.9%) and a secondary hip preservation surgery rate of 8.9% (range 0.0 to 17.4%) in seven 5-year follow-up studies including a total of 873 hips [3]. Again, both reported rates are substantially higher than those found in our series. A detailed comparison of patient-reported outcome measures with recently published studies on hip arthroscopy starting in the central compartment was slightly in favor of the peripheral-first approach and is summed up in Table 2 [20–22].

**Table 2:** Patient-reported outcome scores in this study compared to those in previous studies

Author and year	Study period	Hips (*n*)	Follow-up (years)	VAS pain (0 is best, 10 is worst)	mHHS (100 is best, 0 is worst)	iHOT12 (100 is best, 0 is worst)	Revision hip arthroscopy rate (%)	Conversion to total hip arthroplasty rate (%)	Procedure
Wagner et al. (this study)	2013–2021	704	6.2 (SD 2.1)	2.8 (SD 2.3)	86.2 (SD 13.1)	78.7 (SD 21.8)	2.6	3.7	Peripheral compartment first
Chen et al. 2018 [20]	2008–2011	101	5.5 (SD 0.67)	2.3 (SD 2.3)	82.4 (SD 17.5)	73.9 (SD 22.4)	4.0	13.9	Central compartment first
Öhlin et al. 2020 [25]	2011–2013	254	5.0 (SD n/a)	n/a	n/a	67.2 (SD 27.5)	2.2	13.6 (survivorship 86.4%)	Central compartment first
Domb et al. 2019 [22]	2008–2013	23	5.6 (SD 0.64)	2.7 (SD 2.7)	80.2 (SD 19.1)	67.1 (SD 28.8)	17.4	13	Central compartment first

The data are given as the mean and standard deviation (SD)

*mHHS* modified Harris Hip Score, *iHOT-12* International Hip Outcome Tool with 12 questions, *VAS* visual analog scale for pain

Overall, our results are in accordance with previously published outcome studies of the peripheral-compartment-first technique by other authors, as shown by a single peripheral-compartment-first cohort of 154 patients who were followed up for 2 years [19], a single cohort of 72 hips followed up for 5 years [8], and a non-randomized controlled cohort study of 30 hips that underwent the peripheral-compartment-first technique and 30 hips that underwent the central-compartment-first technique [9]. All of the above-cited studies advocate the use of the peripheral-compartment-first technique, mainly because of a reduced complication rate associated with this technique.

The reason for the reduced complication rate remains unclear. However, hip arthroscopy is a complex surgical procedure with a significant learning curve. Iatrogenic damage to the labrum and cartilage is a common complication. In contrast to arthroscopy of the knee, shoulder, elbow, or ankle, palpation cannot be used to guide portal placement in this procedure, owing to the deep location of this joint. Portal placement may be the most dangerous step in hip arthroscopy in terms of iatrogenic labrum or cartilage damage, and direct visualization of portal placement seems a viable way to prevent damage; however, this has never been demonstrated in the literature. Another benefit of the peripheral-compartment-first technique with separate portals for intra-articular and peripheral arthroscopy might be the lack of large capsulotomies. The literature has shown significant concerns with unrepaired capsulotomies [23], and it may be assumed that small stabbing incisions are superior to large capsulotomies, even if repaired. However, well-designed comparative studies are needed to clarify the clinical significance of these theoretical advantages of the peripheral-compartment-first technique. The peripheral-compartment-first technique is simple and reproducible, and our clinical results are in accordance with previous literature. The peripheral-compartment-first approach is most popular in Europe, in specialized departments with a relatively high number of cases. Data on time efficacy show that the set-up time and procedure time of this technique are very similar to those of standard hip arthroscopy. The set-up in the peripheral-compartment-first technique required 5 min more than the traditional technique (38 min vs. 33 min) [24], and the surgery required only 3 min more (116 min vs. 119 min) than the traditional technique [24]. Overall, there was no seemingly relevant time difference between the two techniques.

Our cohort study, with a large sample size for this surgical technique and a midterm follow-up period of 6 years, adds to the existing literature. The major limitations of this study include a relatively small return rate for the patient-reported outcomes of 60.2%, the lack of a control group to compare this method with hip arthroscopy starting in the central compartment, and the lack of baseline patient-reported outcome assessment before surgery.

## Conclusion

The peripheral-compartment-first technique was associated with improved complication rates, conversion to total hip arthroplasty rates and patient-reported outcomes in comparison with previously reported data for hip arthroscopy starting in the central compartment. We recommend a well-conducted randomized controlled clinical trial to compare those techniques and guide future therapeutic recommendations.

## Data Availability

The datasets generated during and/or analyzed during the current study are not publicly available due to patient confidentiality regulations but are available from the corresponding author on reasonable request.
